# Quantitative evaluation of the role of aging and non-aging factors for predicting threats from major chronic diseases and developing control strategies

**DOI:** 10.1016/j.heliyon.2024.e34224

**Published:** 2024-07-06

**Authors:** Liu Hui

**Affiliations:** College of Medical Laboratory, Dalian Medical University, Dalian, 116044, China

**Keywords:** Chronic disease, Noncommunicable diseases, Mortality, Disease management, Risk assessment

## Abstract

Various indicators exist to assess the threat of chronic diseases. This paper presents new ones to evaluate the role of aging and non-aging factors for predicting threats from major chronic diseases. Age at zero mortality (AM0) and age at average mortality (AMa) can be calculated by regressing age and mortality (the intercept indicates AM0, the slope indicates the observed slope and r indicates random non-aging factors). A regression equation can be created using AMa at the age of 72 and mortality at the age of 82; thus, the expected slope can be obtained for the aging factor without considering non-aging factors. It is possible to distinguish between aging and non-aging factors using the observed and expected slopes, which should be multiplied by r to produce an index of aging (IA). The lower the AM0, AMa or IA of a disease is, the greater the threat it poses to a population. The AM0 and IA were calculated using data from China (2004 and 2019) for various diseases [cancer, heart disease (HD), cerebrovascular disease (CVD), and chronic obstructive pulmonary disease (COPD)]. We found the severity of threat was highest for cancer, CVD, other chronic diseases, HD and COPD in descending order in 2019. The results suggest that changes in threats may be related to socioeconomic development. Cancer was found to be the greatest threat to younger age groups, with IA<0.5, suggesting that non-aging risk factors may play an important role in cancers. Conversely, aging may play an important role in other chronic diseases, including HD, CVD, and COPD. Compared to 2004, the AM0 of cancer showed the greatest change. In conclusions, the different indicators explain different aspects of the problem and it would be beneficial to conduct in-depth research on the theoretical basis for the association of threats of disease with socioeconomic development in order to develop prevention and control strategies.

## 1 Introduction

Chronic diseases have become a major threat to survival and the primary cause of death worldwide, in light of socioeconomic development [[1], [2], [3]]. An increasing elderly population with greater longevity might be important reason for this [[4], [5], [6]]. Nevertheless, there is a tendency toward a reduced incidence for some chronic diseases, such as chronic obstructive pulmonary disease (COPD) [7]. Obviously, different chronic diseases present different threats to survival with improved medical care and increased human longevity in modern society [3,7,8].

The general thinking is that chronic diseases are driven by aging and non-aging factors [[9], [10], [11]]. Aging is a complex process that involves the gradual decline in physiological functions that are necessary for survival and fertility. Aging is an omnipresent variable for all living organisms and a natural part of the life cycle, although the rate at which it occurs can vary significantly between species and even between individuals within a species.

Non-aging factors are those driving factors related to genetics, the environment, and medical treatments. More importantly, strength of aging and non-aging factors also changes with time, such as economic, social, environmental, and human health. Therefore, aging and non-aging factors include many variables that change with time, which is inconsistent with the increasing life expectancy that is occurring within developed societies [3,5,8]. Thus, it is necessary to assess the degree of threat from aging and non-aging factors at different times and the burden that different chronic diseases present to health and survival.

The proportion of people dying of a specific cause (the age-standardized death rate) is commonly used to evaluate the risk of a disease in an affected population [3,8]; however, death among younger age groups is not enough to consider. The potential years of life lost (PYLL) and cause deleted life expectancy (CDLE) are also commonly used to evaluate the threat of disease [[12], [13], [14]]. The World Health Organization (WHO) developed a methodology to quantify the health of a population using summary measures, such as disability-adjusted life years (DALY) [[15], [16], [17], [18]]. When discussing the threat of a chronic disease to survival, factors related to both the total number of deaths and the number of deaths among relatively young people should be taken into consideration. Therefore, it is necessary to simplify the methodology with commonly used data on vital statistics to evaluate the threat of chronic diseases.

We know that chronic diseases are related to age, even just from personal experience; therefore, age could be considered to be as a sensitive marker. In this study, I made a quantitative analysis of the occurrence of different diseases by age as a probe, based on data from the National Disease Mortality Surveillance System in Mainland, China. By comparing different diseases and levels of social development at different times (using data obtained from 2004 to 2019), I established patterns of the occurrence of major chronic diseases as a basis to predict the threat of chronic diseases and assess the effectiveness of current prevention and treatment measures.

## 2 Materials and methods

### 2.1 Original data

The raw data (Table 1, Table 2, Table 3) were obtained from the National Disease Mortality Surveillance System, 2004 and 2019, which was edited by the Chinese Center for DiseaseTable 1, Table 2, Table 3 Control and Prevention [19,20]. These data were obtained from over 73 million people. Underlying causes of death were classified according to the International Classification of Diseases 10th Revision codes [21] to determine mortality statistics. Cancers, heart disease (HD), cerebrovascular disease (CVD), and chronic obstructive pulmonary disease (COPD) account for more than 80 % of chronic diseases in China [7]. Therefore, these four diseases were included in this paper. As acquired immune deficiency syndrome (AIDS) is a sexually transmitted disease that is not related to age, it was used as a control disease to test the validity of the model. The Classification of Diseases 10th Revision's codes [21] were used to determine mortality statistics (cancer: C00-97; HD: I20-25; CVD: I60-69; COPD: J40-44; AIDS: B20-24).

**Table 1:** Age-stratified rates of death from major chronic diseases in the monitored population of China in 2019 (annual deaths per 10^5^ population).

Age groups	Total of chronic diseases	Cancer	HD	CVD	COPD	Other chronic diseases	Survival
55∼	472.69	213.84	75.62	95.18	12.85	75.20	17445735
60∼	728.72	319.53	116.12	154.92	32.08	106.07	17480461
65∼	1283.09	507.25	213.41	301.38	76.53	184.52	14072230
70∼	2323.96	751.84	424.97	606.58	189.85	350.72	8860096
75∼	4131.73	1043.06	848.17	1159.58	427.35	653.57	5795999
80∼	6793.85	1225.45	1638.43	1938.47	818.06	1173.44	4124288
>85	17446.33	1820.59	5245.11	4611.32	2287.25	3482.06	2146285
Total	2131.47	545.20	472.63	551.98	204.56	357.11	69925094
aSM	325.83	95.09	66.67	79.55	26.44	58.08	–

a Standardized mortality: the age-standardized death rates includes all age groups from group 0–5 to group >85 standardized by 2000 in China.

**Table 2:** Age-stratified rates of death from major chronic diseases in the monitored population of China in 2004 (annual deaths per 10^5^ population).

Age groups	Total of chronic disease	Cancer	HD	CVD	COPD	Other chronic diseases	Survival
55∼	754.31	318.48	80.48	177.33	65.56	112.46	2957812
60∼	1164.95	437.71	128.03	295.00	140.06	164.15	2486122
65∼	2013.78	645.69	235.36	563.08	302.40	267.25	2125649
70∼	3550.87	939.85	446.79	1035.82	667.63	460.78	1601637
75∼	5632.75	1156.61	773.22	1743.47	1216.94	742.51	1022733
80∼	9194.30	1333.52	1426.07	2916.09	2248.32	1270.30	545697
>85	13329.57	1286.53	2644.39	3880.01	3405.99	2112.65	308427
Total	2713.99	653.09	376.25	786.10	522.83	375.72	11048077
aSM	445.25	123.90	57.86	119.03	76.21	68.25	–

a Standardized mortality: the age-standardized death rates include all age groups from group 0–5 to group >85, standardized by 2000 in China.

**Table 3:** Age-stratified rates of death from AIDS in the monitored population of China in 2019 (annual deaths per 10^5^ population).

Age groups	Rate of death	Age groups	Rate of death
55∼	0.780	75∼	1.240
60∼	0.860	80∼	0.920
65∼	0.870	>85	0.650
70∼	1.170	–	–

### 2.2 Model of mortality with age

Aging factors include organ failure, decline in the function and number of mitochondria, increased DNA damage, and reduced cellular replication capacity. These changes contribute to the physical signs of aging with time; therefore, the level of aging is marked by age.

In the realm of mortality, one undeniable truth prevails: time is the ultimate arbiter of life's finite nature. While the proximate causes of death may vary, a profound insight emerges: the primary cause of time-dependent deaths could be aging itself. Therefore, a regression equation was used to determine if there was a linear relationship between age (i.e., different age groups) and mortality from different diseases (with mortality as X and age as Y). The correlation coefficient (*r*) gives the impact of random factors (non-aging factors) on a disease, such as an accident of severe environmental pollution, which also play important roles in diseases.

### 2.3 Model of the impact of aging and non-aging factors

The accumulation of damage from non-aging factors over time could also present time-dependent deaths as an aging factor. Thus, it is necessary to distinguish between aging and non-aging factors using analytical models.

**Fig. 1:**
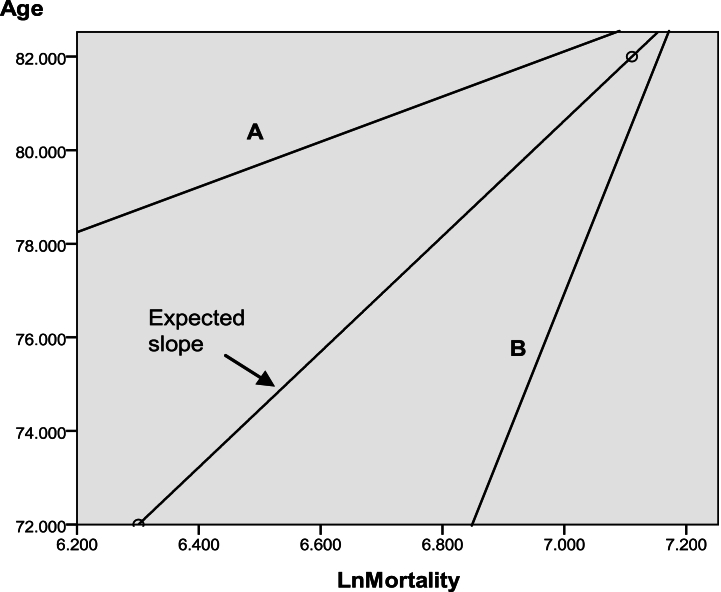
Expected slope and observed slope derived from the analytical model A: Observed slope, which is less than the expected slope, indicating the accumulation of protective effects from non-aging factors. B: Observed slope, which is greater than expected slope, indicating the accumulation of harmful effects from non-aging factors.

The assumption of my model is that aging may play a major role in deaths over 60 years of age, according to the literature [22,23], and a linear relationship should be obtained between age and mortality at a given age. Thus, the expected slope (Se) can be obtained for the aging factor without considering non-aging factors. When non-aging factors are added to aging factors, the Se should change, as shown in Fig. 1. Therefore, it can be used to identify the factors driving mortality that differ between aging and non-aging causes of mortality, by comparing it to the observed slope (So) obtained by the regression equation for each observed mortality in each age group.

The Monte Carlo method was also used to demonstrate the validity of the above model [24]. The association between multiple factors and results is usually explained by a superimposed model, in which aging and non-aging factors should play a role in a superimposed manner that leads to a total effect size. Thus, a simulated dataset was created, as shown in Table 4.

**Table 4:** The simulated dataset of a total effect size of aging and non-aging factors.

Age groups	Effect size			Age groups	Effect size		
	Aging	Non-aging	Total		Aging	Non-aging	Total
55∼	0.7	0.3	1.0	75∼	3.5	1.5	5.0
60∼	1.4	0.6	2.0	80∼	4.2	1.8	6.0
65∼	2.1	0.9	3.0	>85	4.9	2.1	7.0
70∼	2.8	1.2	4.0	–	–	–	–

### 2.4 Establishment of an index of aging

The average mortality (AMa) between 57 and 87 years of age should fall at the median age group (72 years in the present study) and the mortality at 82 years of age (the highest age group with complete data in the present study) can be considered to reflect death from most aging causes. A regression equation and Se can be obtained using AMa at the age of 72 and mortality at the age of 82.

Due to the use of natural logarithms to create equations for mortality rates, the slope should be converted to an exponent for comparability. The ratio of the observed slope (So) to the expected slope (Se) can determine the degree of conformity between the observed value and the expected value. When this ratio value is 1, this indicates complete conformity between the So and Se. When this ratio is > 1 or <1, this indicates the effect of non-aging factors. To make ratio values > 1 and < 1 comparable, this ratio should be converted based on the research literature [25]. In light of the combined effects of cumulative and random non-aging factors, the formula for calculating the index of aging (IA) is as follows:

(1) $$IA=\left\{1-{\left[\left(\frac{\arctan\;e^{So-Se}}{90}-0.5\right)\times 2\right]}^{2}\right\}\times r\;\left(\text{So}>\text{Se}\right)$$

(2) $$IA=2-\left\{1-{\left[\left(\frac{\arctan\;e^{So-Se}}{90}-0.5\right)\times 2\right]}^{2}\right\}\times r\;\left(\text{So}<\text{Se}\right)$$

where r (the correlation coefficient) is derived from linear regression between age and mortality. The range of IA is 0–2. In theory, the IA should be 1.0 when only aging plays a role in death; thus, a range of IA between 0.5 and 1.5 indicates that aging is a major factor in death. It is likely that the threat of a disease is mainly from non-aging risk factors when IA<0.5. Conversely, it can be considered to reflect the effect of comprehensive protection from non-aging factors on a disease when IA>1.5, such as improvements in medical treatments.

### 2.5 Comprehensive assessment of threat from a disease

The age at zero mortality (AM0) is defined as the age at mortality with 10^−5^. The intercept of the linear regression was determined by AM0. AM0 gives greater weight to the threat of a disease for younger age groups by combining the number of cases of a disease in a population with the risk to relatively young people, based on a comprehensive assessment of threat from a disease. AM0 also represents constant non-aging factors. The lower the AM0 of a disease, the greater the threat it poses to a population.

## 3 Results

**Fig. 2:**
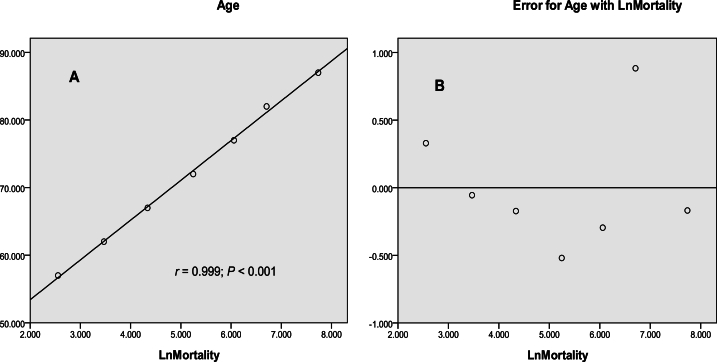
A linear quantitative relation between the median age of the age groups and the logarithm of mortality (A) and residual analysis (B).

**Fig. 3:**
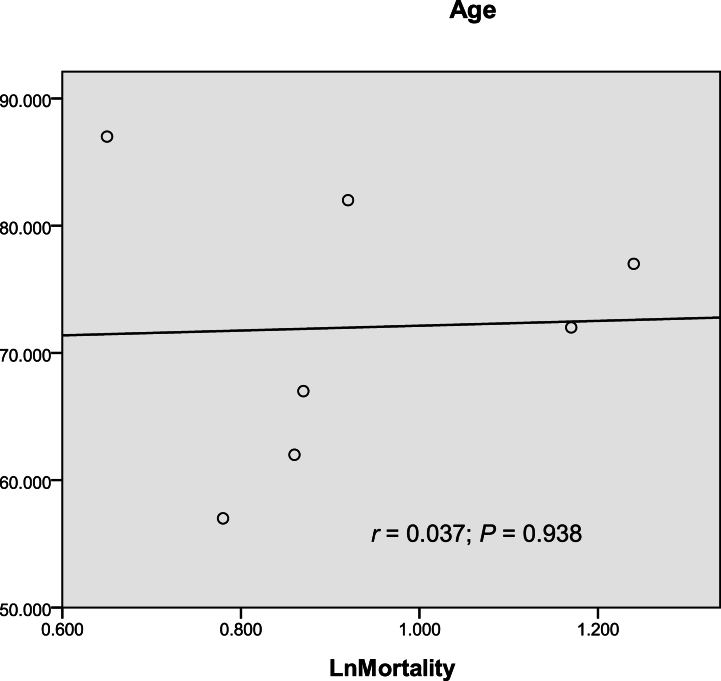
Non-linear relationship between the median age of the age groups and the logarithm of mortality (LnMortality) for AIDS.

A line regression equation was created using the median age of an age group as X and the logarithm of mortality (log mortality) as Y (r = 0.999, p < 0.001). The linear relationship between different age groups and LnMortality, using COPD in 2019 as an example, is shown in Fig. 2(A). The distribution of residuals from the linear regression model was uniformly distributed, as also shown in Fig. 2(B). However, the linear relationship between different age groups and LnMortality was not observed in AIDS, as shown in Fig. 3.

**Fig. 4:**
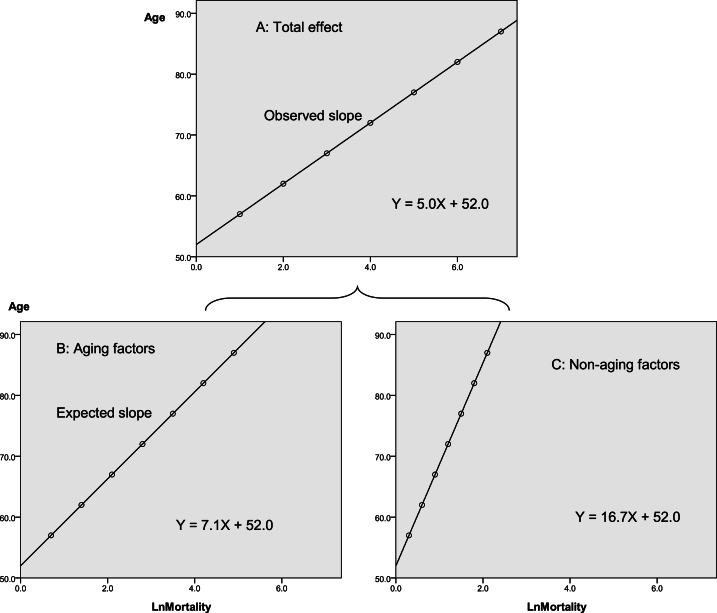
A total effect size (A) of aging factors (B) acted with non-aging factors (C).

The simulated dataset was used to distinguish between aging and non-aging factors, by comparing the observed slope (So) with the Se as shown in Fig. 4. Fig. 4(A–C) showed that observed slope (total effect size) [Fig. 4(A)] increased when aging factor [Fig. 4(B)] and non-aging factor [Fig. 4(C)] acted together.

Age at mortality (AM0 and AMa), R and IA in 2004 and 2019 are shown in Table 5. The IA in both 2004 and 2019 of three diseases (excluding cancer) had almost fallen within the 0.5 to 1.5 range based on Equation (1) and Equation (2), indicating that aging played a major role in deaths from these three diseases. It is worth noting that IA of five diseases (including cancer and other chronic diseases), in 2019 were greater than that in 2004 and M0 in 2019 were also greater than that in 2004, suggesting a tendency toward a reduced effect of damage from non-aging factors on chronic diseases.

**Table 5:** The correlation coefficient (*r*), age at mortality (AMa and AM0) and index of aging (IA) using 2004 and 2019 data.

Diseases	Years	Regression	*r*	AMa	IA	AM0
Cancer	2019	Age = 14.037LnMortality-19.345	0.993	69.104	0.407	−19.345
	2004	Age = 18.424LnMortality-50.573	0.960	68.846	0.029	−50.573
Heart disease	2019	Age = 7.103LnMortality+28.160	0.992	71.902	1.283	28.160
	2004	Age = 8.482LnMortality+20.383	0.999	70.683	0.705	20.383
Cerebrovascular disease	2019	Age = 7.733LnMortality+22.534	0.998	71.356	1.022	22.534
	2004	Age = 9.275LnMortality+8.601	0.996	70.438	0.424	8.601
COPD	2019	Age = 5.886LnMortality+41.643	0.999	72.962	1.450	41.643
	2004	Age = 7.385LnMortality+25.271	0.995	71.496	0.891	25.271
Other chronic diseases	2019	Age = 7.813LnMortality+25.381	0.990	71.306	1.135	25.381
	2004	Age = 10.045LnMortality+10.358	0.999	69.913	0.362	10.358
Total of chronic diseases	2019	Age = 8.428LnMortality+6.295	0.995	70.892	1.020	6.295
	2004	Age = 10.135LnMortality-10.135	0.998	69.994	0.330	−10.135

Threat levels from the five chronic diseases that were investigated by AM0 (the new index) were also compared with standardized mortality (the classic index), as shown in Table 6. The difference in AM0 between 2019 and 2004 was interpreted as a tendency toward an increased or decreased threat from a chronic disease, giving greater weight to the threat of a disease for younger age groups. The values > 1 indicate a protective effect; hence, greater values imply stronger protective effects. The ratio of standardized mortality (2019/2004) was also interpreted as a tendency toward an increased or decreased threat from a chronic disease using the classic index; i.e., lower values imply stronger protective effects. However, the rank order of the five chronic diseases that were investigated was different using the AM0 and standardized mortality.

**Table 6:** Changes of threat from the five chronic diseases investigated between 2004 and 2019 using difference of age at zero mortality (2019–2004) and the ratio of standardized mortality (2019/2004).

Diseases	Age at zero mortality		Standardized mortality	
	Difference^a^	Rank	Ratio^b^	Rank
Cancer	31.228	1	0.767	3
Heart disease	7.777	5	1.152	5
Cerebrovascular disease	13.933	4	0.668	2
COPD	16.372	2	0.347	1
Other chronic diseases	15.023	3	0.851	4
Total of chronic diseases	16.430	–	0.732	–

a Difference (2019–2004): Positive values indicate a protective effect; greater values imply stronger protective effects.

b Ratio (2019/2004): Ratio <1 indicates a protective effect; lower values imply stronger protective effects.

## 4 Discussion

Aging is a complex process and the core of this argument relies on an intrinsic link among aging factors, non-aging factors, and mortality. Aging and non-aging factors are mixed together, making it difficult to use cohort studies. In this study, an analytical model was established to analyze and observe these two factors separately. It is, to my knowledge, the first report of a linear model that quantitatively analyzed the aging and non-aging factors of a disease.

Cancers, HD, CVD, and COPD may account for more than 80 % of chronic diseases in China [7]. Therefore, these four diseases were included in this study using Chinese census information from 2004 to 2019, and **a** linear relationship between different age groups and LnMortality was observed. However, the linear relationship between different age groups and LnMortality was not observed for AIDS. Because AIDS is a sexually transmitted disease that is not related to aging, the AIDs results demonstrate the validity of the analytical method. It was also demonstrated by simulated data that the analytical model was validity.

When discussing the threats for a chronic disease, threats to both a total population and a relatively young population need to be addressed. Therefore, AM0 and AMa were used here to comprehensively analyze threats. The threat to survival from certain diseases was evaluated using the AM0 in 2019, which showed that the most severe threats in 2019 were cancer, CVD, other chronic diseases, HD, and COPD in descending order. Cancer was found to pose a greater threat to relatively young populations, which is consistent with relevant research reports [[26], [27], [28]]. The results also suggest that changes in threats may be related to socioeconomic development.

The IAs based on Se and So could distinguish between aging and non-aging factors using analytical models. The advantage of using IA is that it allows for more accurate estimates by separately focusing on the threat from aging and non-aging factors. When IA < 0.5 or >1.5, it can be assumed that non-aging factors could play an important role in a disease. The results showed that cancer was the greatest threat to younger age groups, with IA < 0.5, but suggest that non-aging risk factors could still play an important role in cancers. Aging could also play an important role in other chronic diseases, including HD, CVD, and COPD. It should be noted that the IA value in 2019 increased, compared with its value in 2004, for all chronic diseases, including cancer, implying that protective factors, such as medical treatments, could play a more important role associated with socioeconomic development and risk factors related to it, such as food additives, consumption of genetically modified foods, and environmental pollution, which might play limited roles. The IAs of five diseases in 2019 were all greater than those in 2004, suggesting there was a tendency toward a reduced effect of harm from non-aging factors on chronic diseases. This is a basis of predicting the threats of major chronic diseases due to socioeconomic development.

When non-aging risk factors play an important role in a chronic disease, the strategy to control this disease should be to improve social and environmental factors. When aging playa an important role in a chronic disease, such as HD, CVD, and COPD in 2019, the strategy to control these diseases should be to improve general health, delaying aging or aging-related deaths. AM0 is a suitable index for evaluating threats to a population from a disease and that IA aids in assessing the roles of aging and non-aging factors for a disease. AM0 and IA can explain different aspects of death from a disease. Obviously, an understanding of the above indicators would be beneficial to find the theoretical basis for more in-depth research on long-lived populations undergoing socioeconomic development, which could lead to the development of effective prevention and control strategies.

Because the role of aging in chronic diseases is more important than non-aging factors; it is reasonable to use age as a probe for assessing basal health status and the impact of disease on individuals. Moreover, it has been suggested for use with experimental indicators to evaluate basal health status and the damage of disease to basal health status [11,29,30], but this method cannot distinguish whether the risk is due to aging or non-aging factors.

There is no effect of the population constituent ratio on that proportion for age-standardized death rates. However, the earlier the age of death is, the greater the impact is on the population; the earlier the age of death is not taken into account for age-standardized death rates. The factors related to both the total population and relatively younger population should be taken into account. AM0 (the new index) is a combination of the number of cases of a disease in a population and the risk to relatively young people; thus, it is a more reasonable index for the comprehensive assessment of threat from a disease. The present results showed that threat levels were different using AM0 and age-standardized death rates (Table 6). It is worth noting that M0 in 2019 were greater than that in 2004 (Table 5), suggesting an increased effect of protective non-aging factors on chronic diseases with socioeconomic development in China.

One of the major limitations of this study is that reasons for the different sensitivity of major chronic diseases to socioeconomic development are not precisely known. However, understanding the above characteristics of chronic diseases is crucial for developing prevention and control strategies. Prevention and control strategies for chronic diseases should take into account the sensitivity of the disease and population to medical conditions and the general health level. For diseases that are sensitive to medical conditions, prevention and control strategies should focus on improving the level of medical treatment. For diseases that are not sensitive to medical conditions, prevention and control strategies should focus on improving the general health level.

## 5 Conclusion

In summary, IA, which is used to analyze death from a disease, allows for a more accurate judgment by separately focusing on the threat from aging and non-aging factors. The AM0, which serves the same purpose, is a combination of the number of cases of a disease and the risk to relatively young people. Given that the proportion of older people in the population will increase because of socioeconomic development, general health status continues to improve, and life expectancy at birth is rising, IA and AM0 are more suitable indicators for assessing the overall risk to survival from chronic diseases. These indicators can explain different aspects of the problem and one can not replace the other. Obviously, an understanding of the above indicators would beneficial for finding the theoretical basis of the association between non-aging factors and disease threats through in-depth research on long-lived populations exposed to socioeconomic development. The hypothesis proposed in this study may aid focused research on the role of cancer, along with socioenvironmental factors, such as quality of life, nutritional status, and lifestyle, on prolonging life.

## Data availability

All data generated or analyzed during this study are included in this published article and no additional data are available.

## Funding

None.

## Authors' contributions

This article has only one author. Liu Hui conceived the analysis and wrote the final version of the manuscript.

## CRediT authorship contribution statement

**Liu Hui:** Writing – original draft, Data curation, Conceptualization.

## Declaration of competing interest

The authors declare the following financial interests/personal relationships which may be considered as potential competing interests:Liu Hui reports was provided by Dalian Medical University. Liu Hui reports a relationship with Dalian Medical University that includes: board membership.
